# BGM-Net: Boundary-Guided Multiscale Network for Breast Lesion Segmentation in Ultrasound

**DOI:** 10.3389/fmolb.2021.698334

**Published:** 2021-07-19

**Authors:** Yunzhu Wu, Ruoxin Zhang, Lei Zhu, Weiming Wang, Shengwen Wang, Haoran Xie, Gary Cheng, Fu Lee Wang, Xingxiang He, Hai Zhang

**Affiliations:** ^1^ Department of Ultrasound, Shenzhen People’s Hospital, The Second Clinical College of Jinan University, Shenzhen, China; ^2^ Department of Gastroenterology, The First Affiliated Hospital of Guangdong Pharmaceutical University, Guangzhou, China; ^3^ Department of Applied Mathematics and Theoretical Physics, University of Cambridge, Cambridge, United Kingdom; ^4^ School of Science and Technology, The Open University of Hong Kong, Hong Kong, China; ^5^ Department of Neurosurgery, Sun Yat-sen Memorial Hospital, Sun Yat-sen University, Guangzhou, China; ^6^ Guangdong Provincial Key Laboratory of Malignant Tumor Epigenetics and Gene Regulation, Sun Yat-Sen Memorial Hospital, Sun Yat-Sen University, Guangzhou, China; ^7^ Department of Computing and Decision Sciences, Lingnan University, Hong Kong, China; ^8^ Department of Mathematics and Information Technology, The Education University of Hong Kong, Hong Kong, China; ^9^ The First Affiliated Hospital of Southern University of Science and Technology, Shenzhen, China

**Keywords:** breast lesion segmentation, boundary-guided feature enhancement, multiscale image analysis, ultrasound image segmentation, deep learning

## Abstract

Automatic and accurate segmentation of breast lesion regions from ultrasonography is an essential step for ultrasound-guided diagnosis and treatment. However, developing a desirable segmentation method is very difficult due to strong imaging artifacts e.g., speckle noise, low contrast and intensity inhomogeneity, in breast ultrasound images. To solve this problem, this paper proposes a novel boundary-guided multiscale network (BGM-Net) to boost the performance of breast lesion segmentation from ultrasound images based on the feature pyramid network (FPN). First, we develop a boundary-guided feature enhancement (BGFE) module to enhance the feature map for each FPN layer by learning a boundary map of breast lesion regions. The BGFE module improves the boundary detection capability of the FPN framework so that weak boundaries in ambiguous regions can be correctly identified. Second, we design a multiscale scheme to leverage the information from different image scales in order to tackle ultrasound artifacts. Specifically, we downsample each testing image into a coarse counterpart, and both the testing image and its coarse counterpart are input into BGM-Net to predict a fine and a coarse segmentation maps, respectively. The segmentation result is then produced by fusing the fine and the coarse segmentation maps so that breast lesion regions are accurately segmented from ultrasound images and false detections are effectively removed attributing to boundary feature enhancement and multiscale image information. We validate the performance of the proposed approach on two challenging breast ultrasound datasets, and experimental results demonstrate that our approach outperforms state-of-the-art methods.

## 1 Introduction

**FIGURE 1:**
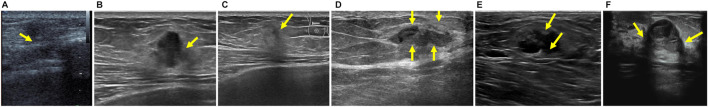
Examples of breast ultrasound images. **(A–C)** Ambiguous boundaries due to similar appearance between lesion and non-lesion regions. **(D–F)** Intensity inhomogeneity inside lesion regions. Note that the green arrows are marked by radiologists.

Breast cancer is the most commonly occurring cancer in women and is also the second leading cause of cancer death Siegel et al. (2017). Ultrasonography has been an attractive imaging modality for the detection and analysis of breast lesions because of its various advantages, e.g., safety, flexibility and versatility Stavros et al. (1995). However, clinical diagnosis of breast lesions based on ultrasound imaging generally requires well-trained and experienced radiologists as ultrasound images are hard to interpret and quantitative measurements of breast lesion regions are tedious and difficult tasks. Thus, automatic localization of breast lesion regions will facilitate the process of clinical detection and analysis, making the diagnosis more efficient, as well as achieving higher sensitivity and specificity Yap et al. (2018). Unfortunately, accurate breast lesion segmentation from ultrasound images is very challenging due to strong imaging artifacts, e.g., speckle noise, low contrast and intensity inhomogeneity. Please refer to Figure 1 for some ultrasound samples.

To solve this problem, we propose a boundary-guided multiscale network (BGM-Net) to boost the performance of breast lesion segmentation from ultrasound images based on the feature pyramid network (FPN) Lin et al. (2017). Specifically, we first develop a boundary-guided feature enhancement (BGFE) module to enhance the feature map for each FPN layer by learning a boundary map of breast lesion regions. This step is particularly important for the performance of the proposed network because it improves the capability of the FPN framework to detect the boundaries of breast lesion regions in low contrast ultrasound images, eliminating boundary leakages in ambiguous regions. Then, we design a multiscale scheme to leverage the information from different image scales in order to tackle ultrsound artifacts, where the segmentation result is produced by fusing a fine and a coarse segmentation maps predicted from the testing image and its coarse counterpart, respectively. The multiscale scheme can effectively remove false detections that result from strong imaging artifacts. We demonstrate the superiority of the proposed network over state-of-the-art methods on two challenging breast ultrasound datasets.

## 2 Related Work

In the literature, algorithms for breast lesion segmentation from ultrasound images have been extensively studied. Early methods Boukerroui et al. (1998), Madabhushi and Metaxas (2002), Madabhushi and Metaxas (2003), Shan et al. (2008), Shan et al. (2012), Xian et al. (2015), Gómez-Flores and Ruiz-Ortega (2016) mainly exploit hand-crafted features to construct segmentation models to infer the boundaries of breast lesion regions, and can be divided into three categories according to Xian et al. (2018), including region growing methods Kwak et al. (2005), Shan et al. (2008), Shan et al. (2012)deformable models Yezzi et al. (1997), Chen et al. (2002), Chang et al. (2003), Madabhushi and Metaxas (2003), Gao et al. (2012), and graph models Ashton and Parker (1995), Chiang et al. (2010), Xian et al. (2015).

Region growing methods start the segmentation from a set of manual or automatic selected seeds, which gradually expand to capture the boundaries of target regions according to the predefined growing criteria. Shan et al. Shan et al. (2012) developed an efficient mehtod to automatically generate region-of-interest (ROI) for breast lesion segmentation, while Kwak et al. Kwak et al. (2005) utilized common contour smoothness and region similarity (mean intensity and size) to define the growing criteria.

Deformable models first construct an initial model and then deform the model to reach object boundaries according to internal and external energies. Madabhushi et al. Madabhushi and Metaxas (2003) initialized the deformable model using boundary points and employed balloon forces to define the extern energy, while Chang et al. Chang et al. (2003) applied the stick filter to reduce speckle noise in ultrasound images before deforming the model to segment breast lesion regions.

Graph models perform breast lesion segmentation with efficient energy optimization by using Markov random field or graph cut framework. Chiang et al. Chiang et al. (2010) employed a pre-trained Probabilistic Boosting Tree (PBT) classifier to determine the data term of the graph cut energy, while Xian et al. Xian et al. (2015) formulated the energy function by modeling the information from both frequency and space domains. Although many a priori models haved been designed to assist breast lesion segmentation, these methods have limited capability to capture high-level semantic features in order to identify weak boundaries in ambiguous regions, leading to boundary leakages in low contrast ultrasound images.

In contrast, Learning-based methods utilize a set of manually designed features to train the classifier for segmentation tasks Huang et al. (2008), Lo et al. (2014), Moon et al. (2014), Othman and Tizhoosh (2011). Liu et al. Liu et al. (2010) extracted 18 local image features to train a SVM classifier to segment breast lesion regions, and Jiang et al. Jiang et al. (2012) utilized 24 Harr-like features and trained Adaboost classifier for breast tumor segmentation. Recently, convolution neural networks (CNNs) have been demonstrated to achieve excellent performance in a lot of medical applications by building a series of deep convolutional layers to learn high-level semantic features from labeled data. Inspired from this, several CNN frameworks Yap et al. (2018), Xu et al. (2019) have been developed to segment breast lesion regions from ultrasound images. For example, Yap et al. Yap et al. (2017) investigated the performance of three networks: a Patch-based LeNet, a U-Net, and a transfer learning approach with a pretrained FCN-AlexNet, for breast lesion detection. Lei et al. Lei et al. (2018) proposed a deep convolutional encoder-decoder network equipped with deep boundary supervision and adaptive domain transfer for the segmentation of breast anatomical layers. Hu et al. Hu et al. (2019) combined a dilated fully convolutional network with an active contour model to segment breast tumors. Although CNN-based methods improve the performance of breast lesion segmentation in low contrast ultrasound images, they still suffer from strong artifacts of speckle noise and intensity inhomogeneity, which typically occur in clinical scenarios, and tend to generate inaccurate segmentation results.

## 3 Our Approach

### 3.1 Overview

**FIGURE 2:**
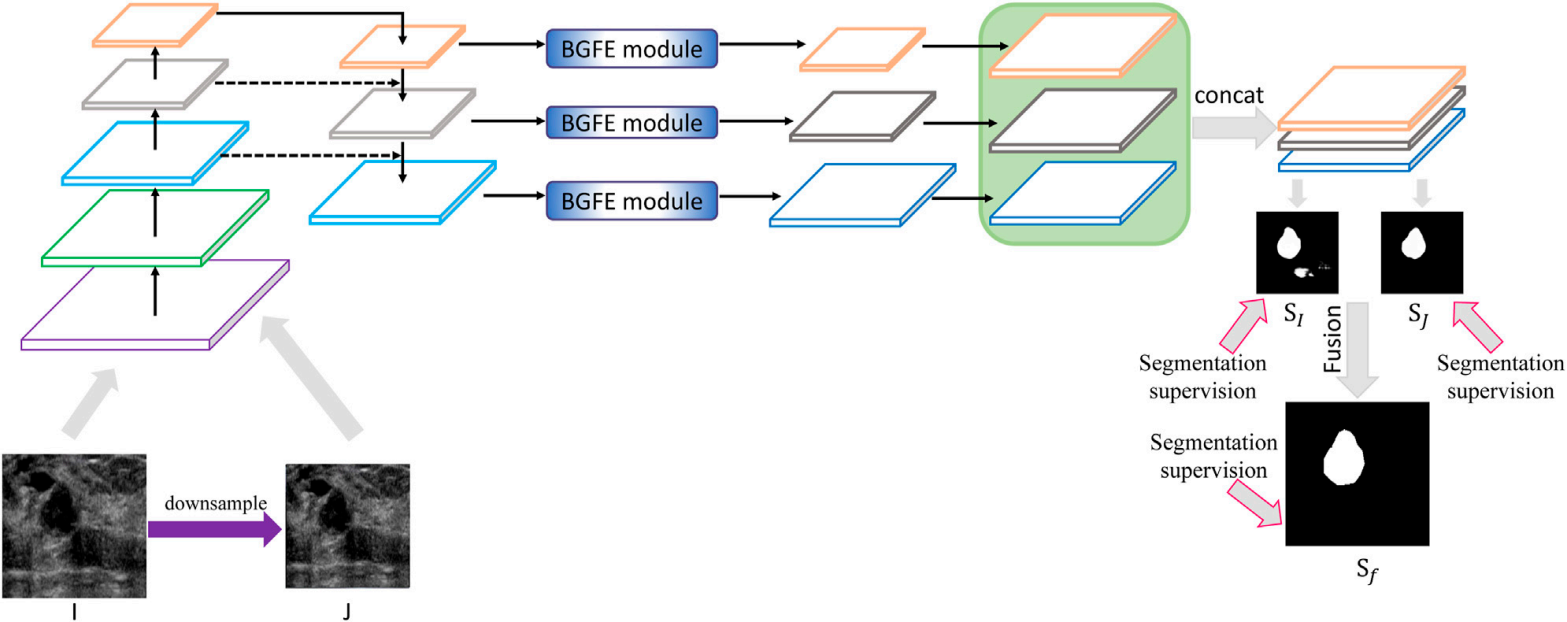
Schematic illustration of the proposed approach for breast lesion segmentation from ultrasound images. Please refer to Figure 3 for BGFE module. Best viewed in color.

Figure 2 illustrates the architecture of the proposed approach. Given a testing breast ultrasound image *I*, we first downsample *I* into a coarse counterpart *J*, and then input both *I* and *J* into the feature pyramid network to obtain a set of feature maps with different spatial resolutions. After that, a boundary-guided feature enhancement module is developed to enhance the feature map for each FPN layer by learning a boundary map of breast lesion regions. All of the refined feature maps are then upsampled and concatenated to predict a fine $S_{I}$ and a coarse $S_{J}$ segmentation maps for *I* and *J*, respectively. Finally, the segmentation result $S_{f}$ is produced by fusing $S_{I}$ and $S_{J}$ so as to leverage the information from different image scales. By combining enhanced boundary features and multiscale image information into a unified framework, our approach precisely segments the breast lesion regions from ultrasound images and effectively removes false detections resulting from various imaging artifacts.

### 3.2 Boundary-Guided Feature Enhancement

The FPN framework first uses a convolutional neural network to extract a set of feature maps with different spatial resolutions and then iteratively merges two adjacent layers from the last layer to the first layer. Although FPN improves the performance of breast lesion segmentation, it still suffers from the inaccuracy of boundary detection because of strong ultrasound artifacts. To solve this problem, we develop a boundary-guided feature enhancement module to improve the boundary detection capability of the feature map for each FPN layer by learning a boundary map of breast lesion regions.

**FIGURE 3:**
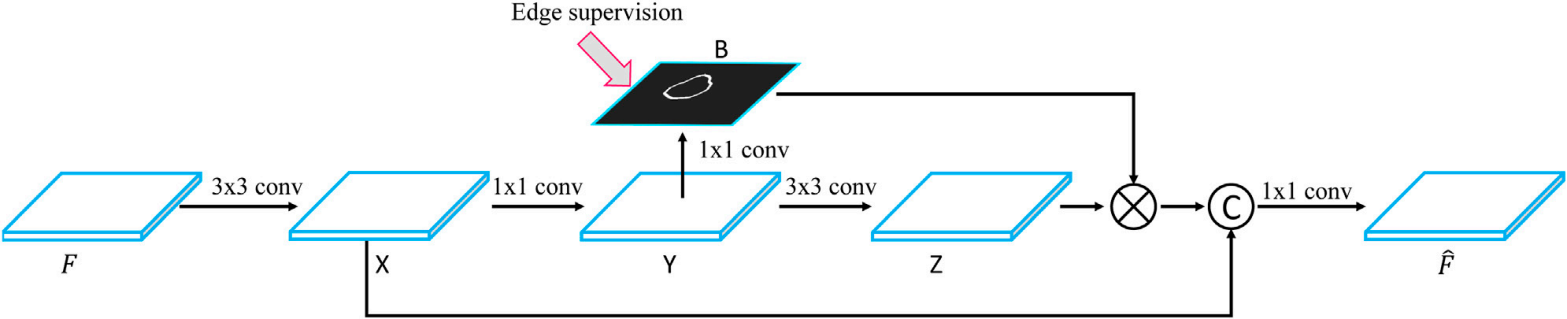
Flowchart of the BGFE module. F and $\hat{F}$ are the feature map and the refined feature map, respectively. Best viewed in color.

Figure 3 shows the flowchart of the BGFE module. Given a feature map *F*, we first apply a 3×3 convolutional layer on *F* to obtain the first intermediate image *X*, followed by a 1×1 convolutional layer to obtain the second intermediate image *Y*, which will be used to learn a boundary map *B* of breast lesion regions. Then, we apply a 3×3 convolutional layer on *Y* to obtain the third intermediate image *Z*, and multiply each channel of *Z* with *B* in an element-wise manner. Finally, we concatenate *X* and *Z*, followed by a 1×1 convolutional layer, to obtain the enhanced feature map $\hat{F}$. Mathematically, the *c*th channel of $\hat{F}$ is computed as:

$${\hat{F}}_{c}=f_{conv}\left(concate\left(\left(Z_{c}\times B\right),X\right)\right)\;,$$ (1)

where $f_{conv}$ is the 1×1 convolutional parameter; $Z_{c}$ is the *c*th channel of *Z*; and concate is the concatenation operation on the feature map.

### 3.3 Multiscale Scheme

After the BGFE module, all of the refined feature maps will be upsampled and concatenated to predict the segmentation map of the input image. To account for various ultrasound artifacts, we design a multiscale scheme to produce the final segmentation result by fusing the information from different image scales. Specifically, for each testing breast ultrasound image, we first downsample it into a coarse counterpart with the resolution of 320×320. In our experiment, the training images are all resized to the resolution of 416×416 according to previous experience, and thus the testing image is also resized to the same resolution. Then, both the testing image and its coarse counterpart are input into the proposed network to predict a fine and a coarse segmentation maps, respectively. Finally, the segmentation result is produced by fusing the fine and the coarse segmentation maps so that false detections from the fine scale can be counteracted by the information from the coarse scale, leading to an accurate segmentation of breast lesion regions.

### 3.4 Loss Fuction

In our study, there is an annotated mask of breast lesion regions for each training image, which will serve as the ground true for breast lesion segmentation. In addition, we employ a canny detector Canny (1986) on the annotated mask to obtain a boundary map of breast lesion regions, which will serve as the ground true for boundary detection. Based on the two ground truths, we combine a segmentation loss and a boundary detection loss to compute the total loss function ℒ as following:

$$ℒ=D_{seg}+\alpha D_{edge}\;,$$ (2)

where $D_{seg}$ and $D_{edge}$ are the segmentation loss and the boundary detection loss, respectively. α is used to balance $D_{seg}$ and $D_{edge}$, and is empirically set to 0.1. The definitions of $D_{seg}$ and $D_{edge}$ are given by:

$$\begin{array}{c} D_{seg}=\hat{\text{Φ}}\left(S_{I},G_{s}\right)+\hat{\text{Φ}}\left(S_{J},G_{s}\right)+\hat{\text{Φ}}\left(S_{f},G_{s}\right)\;,\\ \;and\;D_{edge}=\overset{3}{\underset{k=1}{\sum }}\hat{\text{Φ}}\left(B_{k},G_{e}\right)\;, \end{array}$$ (3)

where $G_{s}$ and $G_{e}$ are the ground truths for breast lesion segmentation and boundary detection, respectively. $S_{I}$ and $S_{J}$ are the segmentation maps of *I* and *J*, respectively, and $S_{f}$ is the final segmentation result. $B_{k}$ is the predicted boundary map of breast lesion regions at the *k*th BGFE module. The function $\hat{\text{Φ}}$ includes a dice loss and a cross entropy loss, and is defined as:

$$\hat{\text{Φ}}={\text{Φ}}_{CE}+\beta {\text{Φ}}_{dice}\;,$$ (4)

where ${\text{Φ}}_{CE}$ and ${\text{Φ}}_{dice}$ are the functions of the cross entropy loss and the dice loss, respectively. β is used to balance ${\text{Φ}}_{CE}$ and ${\text{Φ}}_{dice}$, and is empirically set to 0.5.

### 3.5 Training and Testing Strategies

#### Training Parameters

We initialize the parameters of the basic convolutional neural network by a pre-trained DenseNet-121 Huang et al. (2017) on ImageNet while the others are trained from scratch noise. The breast ultrasound images in our training dataset are randomly rotated, cropped, and horizontally flipped for data augmentation. We use Adam optimizer to train the whole framework by 10, 000 iterations. The learning rate is initialized as 0.0001 and reduced to 0.00001 after 5, 000 iterations. We implement our BGM-Net on Keras and run it on a single GPU with a mini-batch size of 8.

#### Inference

We take $S_{f}$ as the final segmentation result for each testing image.

## 4 Experiments

This section conducts extensive experiments, as well as an ablation study, to evaluate the performance of the proposed approach for breast lesion segmentation from ultrasound images.

### 4.1 Dataset

Two challenging breast ultrasound datasets are utilized for the evaluation. The first dataset (i.e., Al-Dhabyani et al., 2020) is from the Baheya Hospital for Early Detection and Treatment of Womenś Cancer (Cairo, Egypt). BUSI includes 780 tumor images from 600 patients. We randomly select 661 images as the training dataset and the remaining 119 images serve as the testing dataset. The second dataset includes 632 breast ultrasound images (denoted as BUSZPH), collected from Shenzhen People’s Hospital where informed consent is obtained from all patients. We randomly select 500 images as the training dataset and the remaining 132 images serve as the testing dataset. The breast lesion regions in all the images are manually segmented by experienced radiologies, and each annotation result is confirmed by three clinicians.

### 4.2 Evaluation Metric

We adopt five widely used metrics for quantitative comparison, including Dice Similarity Coefficient (Dice), Average Distance between Boundaries (ADB, in pixel), Jaccard, Precision, and Recall. Please refer to Chang et al. (2009), Wang et al. (2018) for more details about these metrics. Dice and Jaccard measure the similarity between the segmentation result and the ground truth. ADB measures the pixel distance between the boundaries of the segmentation result and the ground truth. Precision and Recall compute pixel-wise classification accuracy to evaluate the segmentation result. Overall, a good segmentation result shall have a low ADB value, but high values for the other four metrics.

### 4.3 Segmentation Performance

#### Comparison Methods

We validate the proposed approach by comparing it with five state-of-the-art methods, including U-Net Ronneberger et al. (2015), U-Net++ Zhou et al. (2018), feature pyramid network (FPN) Lin et al. (2017), DeeplabV3+ Chen et al. (2018) and ConvEDNet Lei et al. (2018). For consistent comparison, we obtain the segmentation results of the five methods by the public code (if available) or by our implementation, which is tuned for the best result.

#### Quantitative Comparison

Tables 1, 2 present the measurement results of different segmentation methods on the two datasets, respectively. Apparently, our approach achieves higher values on Dice, Jaccard, Precision and Recall measurements, and lower value on ADB measurement, demenstrating the high accuracy of the proposed approach for breast lesion segmentation from ultrasound images.

**TABLE 1:** Measurement results of different segmentation methods on the BUSZPH dataset. Our results are highlighted in bold.

Method	Dice	ADB	Jaccard	Precision	Recall
U-Net Ronneberger et al. (2015)	0.7819	15.6556	0.6990	0.8055	0.8429
U-Net++ Zhou et al. (2018)	0.7895	11.3389	0.7092	0.8408	0.8029
FPN Lin et al. (2017)	0.8597	5.6913	0.7829	0.9001	0.8518
DeeplabV3+ Chen et al. (2018)	0.8418	6.6364	0.7583	0.8870	0.8289
ConvEDNet Lei et al. (2018)	0.8368	5.7943	0.7540	0.8987	0.8249
Our approach	**0.8688**	**4.7966**	**0.7961**	**0.9080**	**0.8603**

**TABLE 2:** Measurement results of different segmentation methods on the BUSI dataset. Our results are highlighted in bold.

Method	Dice	ADB	Jaccard	Precision	Recall
U-Net Ronneberger et al. (2015)	0.7696	33.4737	0.6777	0.8451	0.7833
U-Net++ Zhou et al. (2018)	0.7622	30.6443	0.6685	0.8222	0.7861
FPN Lin et al. (2017)	0.8267	16.6268	0.7409	0.8479	0.8539
DeeplabV3+ Chen et al. (2018)	0.8268	16.2611	0.7348	0.8720	0.8337
ConvEDNet Lei et al. (2018)	0.8270	17.3333	0.7357	0.8490	0.8551
Our approach	**0.8397**	**12.5637**	**0.7597**	**0.8931**	**0.8345**

#### Visual Comparison

**FIGURE 4:**
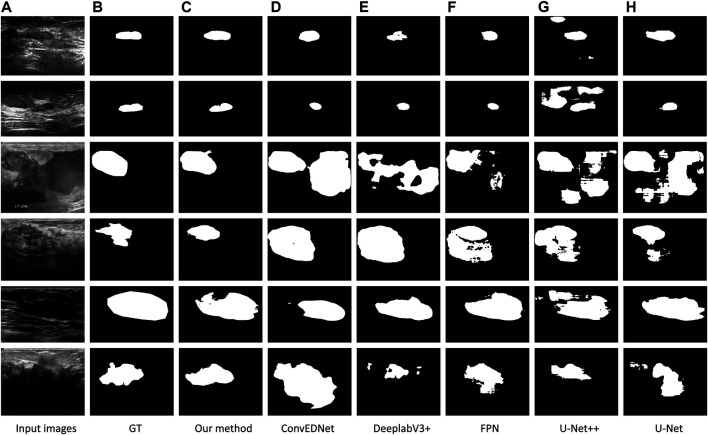
Comparison of breast lesion segmentation among different methods. **(A)** Testing images. **(B)** Ground truth (denoted as GT). **(C–H)**: Segmentation results obtained by our approach (BGM-Net), ConvEDNet Lei et al. (2018), DeeplabV3+ Chen et al. (2018), FPN Lin et al. (2017), U-Net++ Zhou et al. (2018), and U-Net Ronneberger et al. (2015), respectively. Note that the images in first three rows are from BUSZPH, while the images in last three rows are from BUSI.

Figure 4 visually compares the segmentation results obtained by our approach and the other five segmentation methods. As shown in the figure, our approach precisely segments the breast lesion regions from ultrasound images despite of sevious artifacts, while the other methods tend to generate over or under-segmentation results as they wrongly classify some non-lesion regions or miss parts of lesion regions. In the first and second rows where high speckle noise is presented, our result shows the highest similarity against the ground true. This is because the boundary detection loss in our loss function explicitly regularizes the boundary shape of the detected regions using the boundary information in the ground true. In addition, non-lesion regions are greatly removed even though there are ambiguous regions with weak boundaries, see the third and fourth rows, since the multiscale shceme in our approach effectively fuses the information from different image scales. Moreover, our approach accurately locate the boundaries of breast lesion regions in inhomogeneous ultrasound images attributing to the boundary feature enhancement of the BGFE module, see the fifth and sixth rows. In contrast, segmentation results from the other methods are inferior as these methods have limited capability to cope with strong ultrasound artifacts.

### 4.4 Ablation Study

#### Network Design

We conduct an ablation study to evaluate the key components of the proposed approach. Specifically, three baseline networks are considered and their quantitative results on the two datasets are reported in comparison with our approach. The first baseline network (denoted as “Basic”) removes both the BGFE modules and multiscale scheme from our approach, meaning that both boundary feature enhancement and multiscale fusing are disabled and the proposed approach degrades to the FPN framework. The second baseline network (denoted as “Basic + Multiscale”) removes the BGFE modules from our approach, meaning that boundary feature enhancement is disabled while multiscale fusing is enabled. The third baseline network (denoted as “Basic + BGFE”) removes the multiscale scheme from our approach, meaning that multiscale fusing is disabled while boundary feature enhancement is enabled.

#### Quantitative Comparison

Tables 3, 4 present the measurement results of different baseline networks on the two datasets, respectively. As shown in the table, both “Basic + BGFE” and “Basic + Multiscale” perform better than “Basic” by showing higher values on Dice, Jaccard, Precision and Recall measurements, but a lower value on ADB measurement. This clearly demonstrates the benifits from the FPN module and the multiscale scheme. In addition, our approach achieves the best result compared with the three baseline networks, which validates the superiority of the proposed approach by combining boundary feature enhancement and multiscale fusing into a unified framework.

**TABLE 3:** Measurement results of different baseline networks on the BUSZPH dataset. Our results are highlighted in bold.

Method	Dice	ADB	Jaccard	Precision	Recall
Basic	0.8496	6.9231	0.7665	0.8840	0.8553
Basic + Multiscale	0.8578	6.3899	0.7816	0.8853	0.8600
Basic + BGFE	0.8619	6.1084	0.7855	0.9006	0.8602
Our approach	**0.8688**	**4.7966**	**0.7961**	**0.9080**	**0.8603**

**TABLE 4:** Measurement results of different baseline networks on the BUSI dataset. Our results are highlighted in bold.

Method	Dice	ADB	Jaccard	Precision	Recall
Basic	0.8158	13.9902	0.7325	0.8641	0.8253
Basic + Multiscale	0.8246	16.6773	0.7385	0.8831	0.8117
Basic + BGFE	0.8300	12.4873	0.7503	0.8669	0.8329
Our approach	**0.8397**	**12.5637**	**0.7597**	**0.8931**	**0.8345**

#### Visual Comparison

**FIGURE 5:**
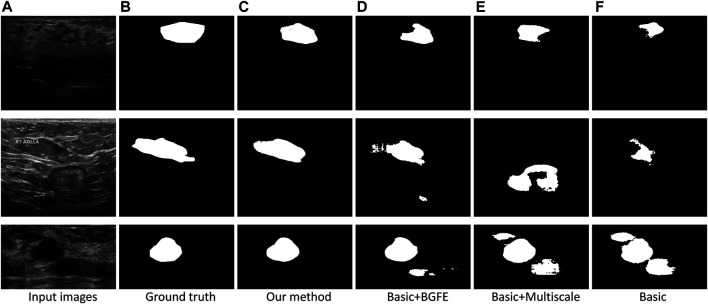
Comparison of breast lesion segmentation between our approach **(C)** and the three baseline networks **(D–F)** against the ground truth **(B)**.

Figure 5 visually compares the segmentation results obtained by our approach and the three baseline networks. Apparently, our approach better segments breast lesion regions than the three baseline networks. False detections resulted from speckle noise are observed in the result of “Basic + BGFE”, while “Basic + Multiscale” wrongly classifies a large part of non-lesion regions due to unclear boundaries in ambiguous regions. In contrast, our approach accurately locates the boundaries of breast lesion regions by learning an enhanced boundary map using the BGFE module. Moreover, false detections are effectively removed attributing to the multiscale scheme. Thus, our result achieves the highest similarity against the ground true.

## 5 Conclusion

This paper proposes a novel boundary-guided multiscale network to boost the performance of breast lesion segmentation from ultrasound images based on the FPN framework. By combining boundary feature enhancement and multiscale image information into a unified framework, the boundary detection capability of the FPN framework is greatly improved so that weak boundaries in ambiguous regions can be correctly identified. In addition, the segmentation accuracy is notably increased as false detections resulted from strong ultrasound artifacts are effectively removed attributing to the multiscale scheme. Experimental results on two challenging breast ultrasound datasets demonstrate the superiority of our approach compared with state-of-the-art methods. However, similar to previous work, our approach also relies on labeled data to train the network, which limits its applications in scenarios where unlabeled data is presented. Thus, the future work will consider the adaptation from labeled data to unlabeled data in order to improve the generalization of the proposed approach.

## Data Availability

The raw data supporting the conclusion of this article will be made available by the authors, without undue reservation. The data presented in the study are deposited in https://sites.google.com/site/indexlzhu/datasets.
